# Eye Movement Desensitization and Reprocessing vs. Treatment-as-Usual for Non-Specific Chronic Back Pain Patients with Psychological Trauma: A Randomized Controlled Pilot Study

**DOI:** 10.3389/fpsyt.2016.00201

**Published:** 2016-12-20

**Authors:** Andreas Gerhardt, Sabine Leisner, Mechthild Hartmann, Susanne Janke, Günter H. Seidler, Wolfgang Eich, Jonas Tesarz

**Affiliations:** ^1^Department of General Internal Medicine and Psychosomatics, University Hospital Heidelberg, Heidelberg, Germany; ^2^Department of Pain Medicine, Berufsgenossenschaftliches Universitätsklinikum Bergmannsheil, Ruhr-University Bochum, Bochum, Germany

**Keywords:** chronic back pain, eye movement desensitization and reprocessing, psychological trauma, treatment

## Abstract

**Objective:**

Eye movement desensitization and reprocessing (EMDR)—an evidence-based approach to eliminate emotional distress from traumatic experiences—was recently suggested for the treatment of chronic pain. The aim of this study was to estimate preliminary efficacy of a pain-focused EMDR intervention for the treatment of non-specific chronic back pain (CBP).

**Design:**

Randomized controlled pilot study.

**Methods:**

40 non-specific CBP (nsCBP) patients reporting previous experiences of psychological trauma were consecutively recruited from outpatient tertiary care pain centers. After baseline assessment, patients were randomized to intervention or control group (1:1). The intervention group received 10 sessions standardized pain-focused EMDR in addition to treatment-as-usual (TAU). The control group received TAU alone. The primary outcome was preliminary efficacy, measured by pain intensity, disability, and treatment satisfaction from the patients’ perspective. Clinical relevance of changes was determined according to the established recommendations. Assessments were conducted at the baseline, posttreatment, and at a 6-month follow-up. Intention-to-treat analysis with last observation carried forward method was used. Registered with http://ClinicalTrials.gov (NCT01850875).

**Results:**

Estimated effect sizes (between-group, pooled SD) for pain intensity and disability were *d* = 0.79 (CI_95%_: 0.13, 1.42) and *d* = 0.39 (CI_95%_: −0.24, 1.01) posttreatment, and *d* = 0.50 (CI_95%_: 0.14, 1.12) and *d* = 0.14 (CI_95%_: −0.48, 0.76) at 6-month follow-up. Evaluation on individual patient basis showed that about 50% of the patients in the intervention group improved clinically relevant and also rated their situation as clinically satisfactory improved, compared to 0 patients in the control group.

**Conclusion:**

There is preliminary evidence that pain-focused EMDR might be useful for nsCBP patients with previous experiences of psychological trauma, with benefits for pain intensity maintained over 6 months.

## Introduction

Chronic back pain (CBP) is common and of socioeconomic relevance (1, 2). For the individual patient, CBP is associated with serious disability and reduced quality of life [e.g., Ref. (1, 3)]. However, for approximately 90% of individuals with CBP, there is no identifiable structural cause (1, 4); these individuals are typically referred to as having non-specific CBP (nsCBP).

Although there are many approaches to treating nsCBP, unfortunately, treatment in these patients has often limited success (5–7) and fails to meet patients’ success criteria (8, 9). This might be the case because many of nsCBP patients report high emotional distress (10–13), but particularly for nsCBP patients with high degrees of emotional distress, classic pain-psychotherapeutic approaches are often insufficient (14). One explanation might be that psychotherapeutic attention paid to patients affected by pain had long focused mainly on cognitive-behavioral factors such as dysfunctional coping strategies and maladaptive behavioral patterns (14). This neglected the fact that emotional distress, e.g., caused by psychological trauma, can also have a central impact on the sensation and processing of pain (15–17). It is well known that physical pain alongside the purely sensory experience of pain generally also comes with a significant emotional dimension (17, 18). This emotional dimension not only influences fundamental aspects such as how severe or distressing a pain is felt to be but also significantly influences the persistence of the pain symptoms (19, 20). Recent studies show that in the context of pain chronification, there is a shift away from the classic pain-processing regions in the brain toward the emotional networks (21). This emotional shift is blamed for the fact that pain may persist in patients with high emotional distress (21). The acknowledgment that pain can become chronic through maladaptive emotional processing forms the pathophysiological basis for applying eye movement desensitization and reprocessing (EMDR) (22) in treating chronic pain.

Eye movement desensitization and reprocessing is a psychotherapeutic approach that was originally developed to eliminate emotional distress resulting from traumatic memories. The EMDR intervention follows a standardized treatment protocol (22) and aims to process dysfunctionally stored disturbing memories and associated cognitions, emotions, and physical sensations (e.g., pain). EMDR has now become an empirically validated and recommended first-line treatment for posttraumatic stress disorders (PTSD) and other conditions that are specifically related to (emotional) stress [e.g., Ref. (23–26)]. The EMDR intervention, as an established procedure for exposing emotional response from trauma therapy, can be used specifically to process emotional distress in patients suffering from chronic pain with the clear objective of processing this dysfunctional emotional shift.

Therefore, preliminary studies (case reports and case series) have used EMDR to treat chronic pain and have indicated that EMDR is able to clinically significantly reduce pain intensity and disability in patients with various pain disorders (27). Furthermore, EMDR improves pain-coping abilities and facilitates relaxation, which reduces pain and pain-related attitudes and beliefs (28). Therefore, EMDR treatment is a promising approach for nsCBP patients, where psychosocial factors seem to be of special significance (e.g., patients with psychological trauma). However, although nsCBP is one of the most frequent types of pain, only one case series used EMDR for nsCBP. This case series showed that EMDR is able to clinically reduce pain intensity and pain interference in nsCBP patients (29).

Therefore, we developed an EMDR manual for nsCBP patients (30) and conducted a randomized controlled pilot study of 10-session manualized outpatient pain-focused EMDR treatment for nsCBP patients with previous experience of psychological trauma (nsCBP-t). The objective was to estimate preliminary efficacy of a pain-focused EMDR intervention in addition to treatment-as-usual (TAU) compared to TAU alone in nsCBP-t patients. Outcomes were pain intensity, disability, and patients’ global impression of change (patient success criteria). In addition, we explored the feasibility (acceptability of randomization, adequate retention rate, and recording of negative effects) to conduct a larger trial.

## Materials and Methods

### Study Design

The study was a mono-center, single-blind (outcome assessor), and two-group parallel randomized controlled pilot study of adult patients with nsCBP-t. The study design has been published elsewhere (31), and the trial protocol was registered with http://ClinicalTrials.gov (NCT01850875). The study was part of the research consortium LOGIN, funded by the German Federal Ministry of Education and Research (01EC1010A) (32). Ethics approval was received from the Ethics Committee Heidelberg (approval No. S-261/2010), and the study was conducted in accordance with the Declaration of Helsinki in its present form. All the participants provided written informed consent.

### Participants

Participants were consecutively recruited (April 2013 to December 2014) from a specialized outpatient clinic for chronic pain disorders at the Department of General Internal Medicine and Psychosomatics of the University Hospital Heidelberg.

The inclusion criteria were as follows: presence of nsCBP, ≥18 years, adequate German language skills, and previous exposure to at least one psychologically traumatic event. The exclusion criteria were as follows: application for retirement pension pending, ongoing psychotherapy, severe dissociation (as dissociation is a known contraindication for EMDR), and severe comorbidity or disorders requiring inpatient care (e.g., anorexia nervosa or severe psychiatric comorbidity).

To confirm the diagnosis of nsCBP, all patients received a physical examination (general, rheumatological, orthopedic, and neurological), with special attention paid to findings that indicated a specific origin of back pain. Therefore, “red flags” [hints of the presence of serious pathology according to the Agency for Health Care Policy and Research Low Back Guidelines (33)] were considered, and attention was paid to the German guidelines for the management on back pain (34). Furthermore, former medical reports and discharge letters were taken into account when available, and all the patients were questioned about their past medical history and their comorbidities. In cases with signs of serious pathological findings, participants were excluded from the current study. Chronicity of back pain was defined as at least 45 days with back pain within the last 3 months. Clinical evaluation was carried out by two physicians with extensive experience with the diagnosis and management of chronic pain conditions, especially musculoskeletal pain.

The experience of psychological trauma was assessed by the Structured Clinical Interview for the Diagnostic and Statistical Manual for Mental Disorders (35). As EMDR is specifically directed to eliminate emotional distress that results from traumatic events, we focused on psychological trauma from specific events. The legal definition of emotional distress states that severe emotional distress exists where a reasonable person normally constituted would be unable to cope adequately with the mental stress engendered by the circumstances of the event (36). Therefore, patients who experienced, witnessed, or were confronted with an event that was accompanied by the involvement of strong negative emotions such as intense fear, helplessness, or horror were classified as subjects having experienced psychological trauma.

The exclusion criteria dissociation was measured by the FDS-20, a German adaption of the Dissociative Experience Scale (37). Patients with scores >12 were excluded.

### Randomization, Masking, and Methodological Aspects

Patients who were eligible and provided written informed consent to participate were enrolled in the study, and baseline data were collected. After acquisition of baseline data, participants were randomly assigned (blocked randomization, 1:1, block length 4) to the intervention group (TAU + EMDR) or control group (TAU only). An independent person randomized each patient using a computer-based system and mailed a fax to the group of therapists.

The outcome assessor (study nurse) was not involved in treatment or randomization and thus was blinded in regard to group membership. Data were managed and analyzed by Andreas Gerhardt.

The EMDR treatment was provided by EMDR-trained physicians (Susanne Janke and Jonas Tesarz) and an EMDR-trained psychologist (Sabine Leisner) who had participated in an EMDR International Association (EMDRIA)-approved basic training program. The therapists were supervised regularly (at least two times per patient) by an EMDRIA-approved consultant (Günter H. Seidler). Before starting the study, a treatment manual was developed to guarantee standardized treatment (30). Each therapist underwent 3-day training on the manual.

Because this was a pilot study, there was no formal sample size calculation. Therefore, estimated effect sizes must be considered as preliminary. Funding was granted for 20 patients in each study group.

### Interventions

All the participants (intervention and control group) received TAU. TAU was a treatment approach following the German disease management guideline “low back pain” (34). This guideline gives structured clinical decision-making aids for providing evidence-based medical care in the German health-care system. This may include patient education, general support and advice, physiotherapy, and simple analgesics according to symptoms. TAU was delivered by our tertiary care pain center and the patients’ general practitioners.

Participants who were allocated to the intervention group received, in addition to TAU, a manualized and 10-session outpatient psychotherapeutic EMDR intervention (every 2 weeks for 90 min). Treatment was delivered at the University Hospital Heidelberg. The treatment manual (30) that was developed for this study was based on the principles of EMDR standard procedure (22) and incorporated established EMDR pain protocols focusing on chronic pain patients (28, 38).

The EMDR procedure combines the use of well-established psychotherapeutic methods (including imaginal exposure and cognitive and self-control techniques) and the use of specific EMDR elements such as bilateral sensory stimulation (e.g., left–right eye movements or bilateral hand-tapping induced by the therapist’s fingers) and the dual focus of attention principle (39). With the dual focus of attention principle, patients simultaneously focus on distressing memories and an external bilateral sensory stimulus. This EMDR procedure is suggested to facilitate information processing of emotionally distressing memories (e.g., traumatic events or pain sensations) and thereby cause a decreasing or even an elimination of the emotional distress related to these memories. The goal of EMDR is to greatly decrease or eliminate emotional distress related to a specific memory (“target”). This typically results in modifications of pain, mood, behavior, and improved coping abilities (28). The possible targets for EMDR processing were disturbing memories, current pain perceptions, and anticipated future painful situations together with the associated cognitions, emotions, and bodily sensations.

According to the study manual (30), the EMDR condition started with two sessions dedicated to treatment planning and preparation (including comprehensive assessment of the patient’s history to identify relevant traumatic and pain-related memories causing emotional distress and dysfunctional emotional response, discussing the patient’s explanatory model, and subsequently providing psychoeducation to develop a better understanding of the links between trauma, pain, emotional response, and the principles of EMDR).

The preparatory sessions were followed by seven desensitizing and reprocessing sessions, started by desensitizing and reprocessing the emotionally most distressing memories; afterward, all pain-associated memories of subsequent events were focused until the subjective degree of distress of these memories dropped down. After distressing memories and thoughts were processed, current pain sensations were focused. In the last session (session 10), future pain issues as future pain crises or potential pain triggers were targeted by installation and reinforcement of EMDR-based skills to cope with future distressing or painful events. According to the standard EMDR procedure, each EMDR session was structured as follows: each session started with a 15-min introduction during which patients were asked about their experiences of the last session and the course since the last session. Afterward, the patient was instructed to recognize a picture that represents the most distressing part of the chosen memory, a negative unreasonable self-belief related to the picture, a positive adaptive cognition, as well as the related emotions and the associated body sensations. Thereupon, the patient was guided to focus on the chosen picture and related feelings/affects, cognitions, and body sensations in brief sequential sets while simultaneously focusing on the external bilateral sensory stimulus given by the therapist. After each set, the patient reported any new associations and body sensations that may have emerged. Such associations and body sensations, generally, became the focus of the next set of double attention. This procedure continued until the target memory and/or unpleasant body sensation was desensitized. After that, more bilateral sensory stimulation was used, while the patient was thinking of a recognized adaptive belief or pleasant body sensation. This was repeated until the new statement felt proper to the patient and until all physical discomfort was dissipated.

If the processing of the traumatic or pain-related distressing event was not completed in a single session, the last 5–10 min of each session were used to assist the patient in returning to a more secure and balanced mental state in using an individual self-calming or relaxation techniques that were designed to bring emotional stability and tranquility. The control group received no specific treatment in addition to TAU.

### Measures

Age, sex, partnership status, education, working status, history of back pain, days with pain within the last 4 weeks, and medication were captured by a questionnaire. Adverse and severe adverse events were documented.

### Outcome Measures

The primary outcome was preliminary efficacy of EMDR treatment after 10 treatment sessions (about 6 months). Following established recommendations for outcome measures in clinical chronic pain trials and recommendations for interpreting clinical importance and the clinical importance of group differences [Initiative on Methods, Measurement, and Pain Assessment in Clinical Trials, IMMPACT (40–43)], we measured pain intensity, disability, and patients’ global impression of change due to treatment. We also determined proportion of patients with clinically relevant changes according to the established recommendations. To measure outcomes, we used instruments suggested by IMMPACT (40).

To assess the primary outcome, participants completed a questionnaire after inclusion in the study and before randomization and treatment allocation (T_0_: baseline) and 2 weeks after study completion (T_1_: after treatment, primary endpoint; at average 6 months after T_0_). There was also a follow-up 6 months after the end of treatment (T_2_, see below).

*Pain intensity*: mean pain intensity within the past 4 weeks was measured using the Numerical Rating Scale (NRS), which ranges from 0 “no pain” to 10 “worst pain imaginable.” We also determined number of individuals in the intervention group with clinical relevant reductions (≥30% = clinically moderately important; ≥50% = substantial change) (41). The NRS is a valid and reliable 1-item instrument that is sensitive to change (44, 45) and recommended by IMMPACT (40).

*Disability*: the German version of the West Haven-Yale Multidimensional Pain Inventory (MPI-D) was used to assess pain-related life interference (46, 47). The subscale consists of 10 questions regarding different aspects of daily life (e.g., interference in social activities, work, daily activities, household chores, family activities) that are rated on a 7-point scale ranging from 0 “no interference” to 6 “extreme interference.” For the interference score, the mean of the 10 items was used (0–6). We also determined the number of individuals in the intervention group with a clinical meaningful change (improvement of ≥0.6) (41). The MPI-D is a reliable and valid instrument (46, 47). Cronbach’s α in the current study was excellent (α_T0_ = 0.93, α_T1_ = 0.95). The MPI is recommended by IMMPACT (40).

*Patients’ perspective of change*: global ratings of change of the overall situation due to treatment was evaluated using the Patient Global Impression of Change (PGIC) scale, which is a single-item rating on a 7-point scale ranging from “very much improved” to “very much worse” (48). Changes of “much improved” and “very much improved” are considered as clinically relevant (41). The PGIC is recommended by IMMPACT (40).

The secondary outcome was feasibility for conducting a larger trial, which was defined as follows: acceptability of randomization ≥80%, retention rate ≥80% in the intervention group (including complete data at end of intervention), and safety. Safety was defined as no recordings of important negative effects (e.g., pain intensity after EMDR intervention >pain intensity before EMDR intervention).

We determined group differences between intervention group and control group at T_1_. Data at T_2_ were compared exploratively.

### Statistical Analyses

All collected data were analyzed using SPSS for Windows 22.0 (SPSS, Inc., Chicago, IL, USA). We analyzed the full analysis set according to the intention-to-treat approach using the last observation carried forward method. Descriptive statistics are presented as means (M) and SDs for continuous variables and absolute numbers (*N*) and percentages for categorical variables. Independent *t*-tests were used for the comparison of between-group differences for continuous variables, and Fisher’s exact test was used for between-group comparisons of categorical data (baseline data). For the primary outcome, preliminary effect sizes and their 95% confidence interval were calculated using Cohen’s *d* for continuous variables (between-group effect sizes, pooled SD). In addition, exploratory data analysis that calculated appropriate summary measures for empirical distribution was performed, and descriptive two-sided *P*-values were calculated. Between-group differences—scores at T_1_ and T_2_—were analyzed using independent *t*-tests.

With regard to secondary endpoint, feasibility, acceptability of randomization, and retention rate were reported descriptively. In addition, negative effects of treatment were reported.

## Results

**Figure 1:**
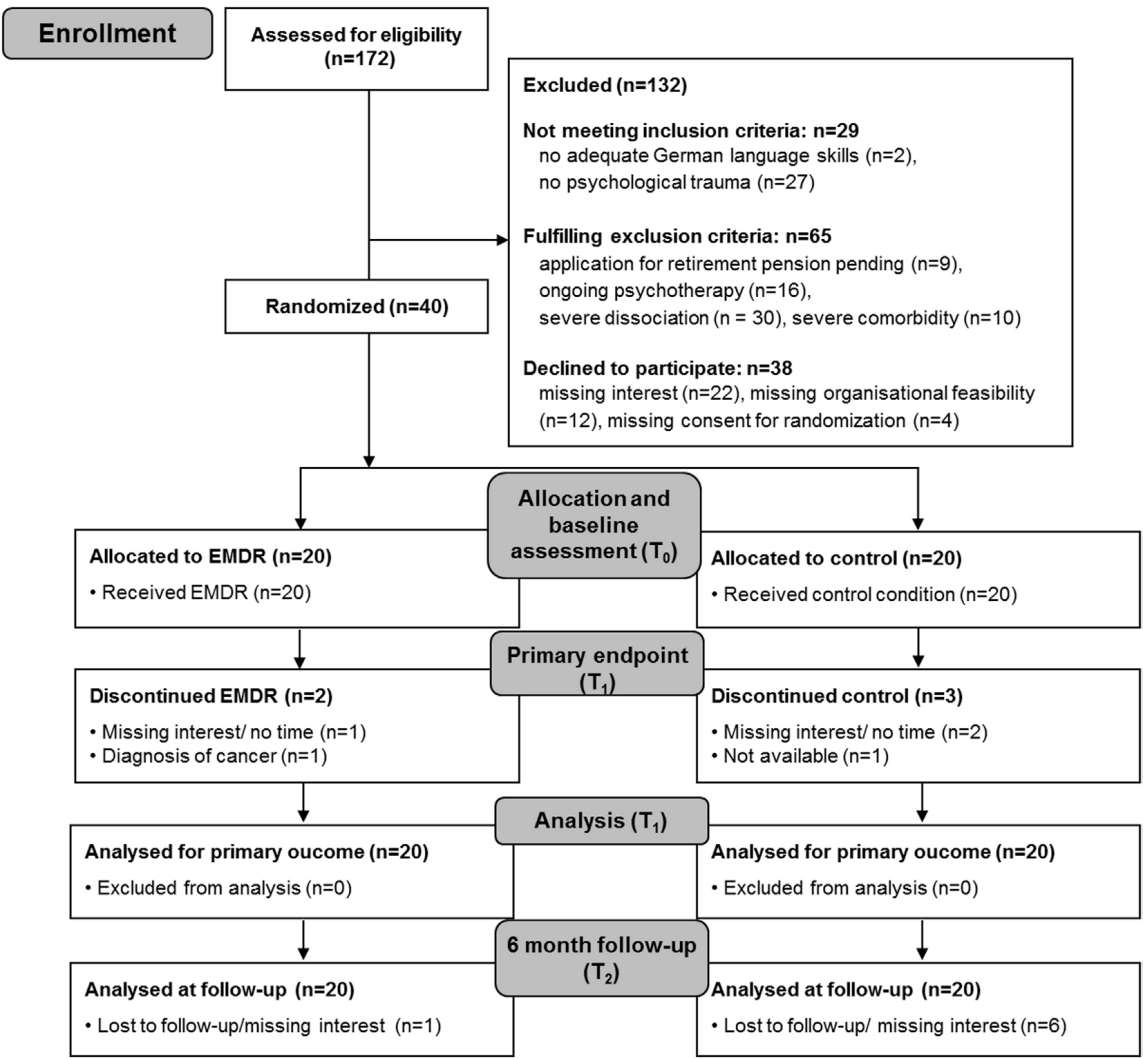
**Participation flow**. EMDR, eye movement desensitization and reprocessing; T_0_, baseline assessment; T_1_, assessment after intervention; T_2_, 6-month follow-up assessment.

We screened 172 patients for eligibility. The main exclusion criterion was the lack of reporting psychological trauma. Acceptability of randomization was 91%. Finally, 40 patients underwent randomization after baseline assessment (T_0_). Twenty patients were randomized to the intervention group and 20 to the control group. At T_1_, two patients in the intervention group and three in the control group had dropped out of the study (dropout rates were 10.0 and 15.0%, respectively). Thus, the retention rate was 90.0% in the intervention group (including complete data at the end of the intervention) and 85.0% in the control group. The two dropouts in the intervention group occurred within treatment session 2 due to time constraints and diagnosis of cancer, respectively. In the control group, two patients refused T_1_ evaluation because of time constraints, and a third patient could not be contacted. In the intervention group, all patients who completed the study attended all the 10 treatment sessions, indicating good treatment adherence. The participation flow chart is presented in Figure 1.

Considering adverse events, the most frequently reported side effects were intense pain during EMDR treatment and short-term increases in pain and tiredness directly following the sessions. These symptoms disappeared after the treatment session or within the following hours. No increases in medication or severe adverse events were reported during the course of treatment.

### Baseline Assessment

All patients were Caucasian. Twenty-eight (70%) patients were females and 12 (30%) were males. Age ranged from 37 to 71 years (M = 56.6, SD = 8.0). The patients suffered from pain for 2 years up to 50 years, with most patients suffering from pain for >10 years. There was no statistical significant difference for any of the baseline variables (age, gender, status of partnership, education, working status, days with pain, pain intensity, history of CBP, number of painful areas, interference, and medication) between the study groups. For the baseline data (sociodemographic data and pain measures) of the intervention group and control group, see Table 1.

**Table 1:** **Sociodemographic characteristics, pain assessment, and psychological variables at baseline (T_0_)**.

	Intervention group	Control group	*T*/Chisq	DF	*P*
Number of subjects	20	20			
Age, M ± SD	56.65 ± 8.88	56.45 ± 7.28	−0.078	38	0.938
Female sex, %	14 (70%)	14 (70%)	0.000	1	1.000
Partnership (firm relationship), *N* (%)	14 (70%)	17 (85%)	1.290	1	0.451
Education (>10 years in school), *N* (%)	6 (30%)	9 (45%)	0.960	1	0.514
Working status, *N* (%)					
Employed	13 (65%)	12 (60%)	1.264	3	1.000
Unemployed	0 (0%)	1 (5%)			
Retired	6 (30%)	6 (30%)			
Others	1 (5%)	1 (5%)			
Pain assessment					
Days with pain last 4 weeks (0–28), M ± SD	26.95 ± 4.70	25.95 ± 5.13	−0.643	38	0.524
CBP intensity (NRS 0–10), M ± SD	4.93 ± 2.41	5.65 ± 1.66	1.108	38	0.275
History of CBP, *N* (%)					
<1 year	0 (0%)	1 (5%)	1.039	2	1.000
1–10 years	8 (40%)	7 (35%)			
>10 years	12 (60%)	12 (60%)			
Number of painful areas (0–10), M ± SD	6.45 ± 2.31	5.70 ± 1.75	−1.159	38	0.254
MPI interference (0–6), M ± SD	2.83 ± 1.57	2.98 ± 1.38	0.324	38	0.747
Pain medication, *N* (%)	11 (55%)	11 (55%)	0.000	1	1.000

*T/Chisq, test statistic; DF, degrees of freedom; P, P values; M, mean; CBP, chronic back pain; NRS, Numerical Rating Scale; MPI, West Haven-Yale Multidimensional Pain Inventory*.

Considering medication, approximately half of the patients in both groups used pain medication, mostly on demand. Two patients in the control group used long-term medication, one used tapentadol (opioid) and one used ibuprofen (non-steroidal anti-inflammatory drug). Medications on demand were non-steroidal anti-inflammatory drugs (diclofenac, ibuprofen, and aspirin) and non-acidic analgesics (acetaminophen and metamizole). One patient in the intervention group used a centrally acting non-opioid analgesic (flupirtine) on demand. There was no significant group difference for any of the measured variables at baseline (T_0_).

### Primary Outcome: Between-Group Comparisons at Posttreatment (T_1_)

*Pain Intensity*: the test of group differences at T_1_ between the intervention group and control group revealed a moderate to large estimated effect size (*d* = 0.79; CI_95%_: 0.13, 1.42). According to recommendations, nine (45%) patients in the intervention group could be classified as clinically relevant improved (improvement ≥30%). Of these, seven showed a substantial (≥50%) and two a moderate improvement (≥30%) (41).

*Disability*: the test of group difference at T_1_ revealed a moderate effect size (*d* = 0.39, CI_95%_: −0.24, 1.01). According to the established recommendations, 10 patients (50%) in the intervention group could be classified relevant improved with a decrease of at least 0.6 points (41).

*Treatment satisfaction from the patients’ perspective*: 10 patients (50.0%) in the intervention group rated their overall alterations during the study phase as much improved (*n* = 8) or very much improved (*n* = 2). In the control group, there were no patients who rated their satisfaction as much or very much improved (*d* = 1.69, CI_95%_: 0.94, 2.38).

For an overview of primary outcomes (T_1_), see Table 2.

**Table 2:** **Pain assessment and psychological variables after treatment (T_1_)**.

	Intervention group	Control group	*T*/Chisq	DF	*P*	*d*
Number of subjects	20	20				
Days with pain last 4 weeks (0–28), M ± SD	23.60 ± 8.01	25.50 ± 5.42	0.878	38	0.386	0.28
CBP intensity (NRS 0–10), M ± SD	3.88 ± 2.15	5.45 ± 1.82	2.499	38	0.017	0.79
MPI interference (0–6), M ± SD	2.09 ± 1.44	2.70 ± 1.65	1.246	38	0.220	0.39
Patient Global Impression of Change (1–7), M ± SD	2.63 ± 1.07	4.18 ± 0.73	5.307	34	0.000	1.69

*T/Chisq, test statistic; DF, degrees of freedom; P, P-values; d, Cohen’s d (positive effect sizes indicate better outcome in intervention group); M, mean; CBP, chronic back pain; NRS, Numerical Rating Scale*.

Considering TAU, patients in the control group had on average 6.0 additional appointments until T_1_ compared to 3.0 appointments in the intervention group (in addition to the initial appointment in our tertiary care pain center at T_0_). Most often, patients were prescribed physiotherapy (40 vs. 15%) and massage (35 vs. 25%) with higher rates in the control group compared to the intervention group.

### 6-Month Follow-up: Between-Group Comparisons (T_2_)

*Pain intensity*: the test of group differences at T_2_ between the intervention group and control group revealed a moderate estimated effect size (*d* = 0.50; CI_95%_: 0.14, 1.12). According to recommendations, 13 (65%) patients in the intervention group could be classified as responders (improvement ≥30%). Of these, five showed a substantial (≥50%) and eight a moderate improvement (≥30%) (41).

*Disability*: the test of group difference at T_2_ revealed a small effect size (*d* = 0.14, CI_95%_: −0.48, 0.76). According to the established recommendations, 7 patients (35%) in the intervention group could be classified as responders with a decrease of at least 0.6 points (41).

*Treatment satisfaction from the patients’ perspective*: eight patients (40.0%) in the intervention group rated their overall alterations during the study phase as much improved (*n* = 4) or very much improved (*n* = 4). In the control group, there were no patients who rated their satisfaction as much or very much improved (*d* = 1.21, CI_95%_: 0.51, 1.86).

For details, see Table 3.

**Table 3:** **Pain assessment and psychological variables after 6-month follow-up (T_2_)**.

	Intervention group	Control group	*T*/Chisq	DF	*P*	*d*
Number of subjects	20	20				
Days with pain last 4 weeks (0–28), M ± SD	23.95 ± 8.64	24.55 ± 7.10	0.240	38	0.812	0.08
CBP intensity (NRS 0–10), M ± SD	3.80 ± 2.24	4.90 ± 2.17	1.574	38	0.124	0.50
MPI interference (0–6), M ± SD	2.19 ± 1.60	2.40 ± 1.34	0.447	38	0.658	0.14
Patient Global Impression of Change (1–7), M ± SD	2.74 ± 1.24	3.94 ± 0.66	3.691	34	0.001	1.21

*T/Chisq, test statistic; DF, degrees of freedom; *P, P*-values; *d*, Cohen’s *d* (positive effect sizes indicate better outcome in intervention group); M, mean; CBP, chronic back pain; NRS, Numerical Rating Scale*.

In addition to the appointment at T_1_ in our tertiary care pain center that was used to adjust TAU, until T_2_ the control group had on average 11 additional appointments compared to 10 appointments in the intervention group. Considering specific interventions, 80 vs. 55% engaged in physiotherapy, 55 vs. 20% in massage, and 45 vs. 10% in psychotherapy for control group compared to intervention group, respectively. This shows that in the control group, more often interventions were prescribed. Moreover, although almost half of the patients in the control group started with psychotherapy, there was a moderate effect of EMDR intervention compared to the control group in favor of EMDR.

## Discussion

In this study, we describe the results of a manualized, 10-session EMDR intervention for the treatment of nsCBP-t patients. Comparisons of pain intensity and disability after treatment between the intervention and control group suggested improvements in the intervention group with moderate to large effect sizes. As recommended, complementary to analysis of between-group effects, we also analyzed the individual response to treatment (42). Our results demonstrated a clinical relevant improvement in pain intensity in nine (45%) patients of the intervention group. Considering disability, a clinical important improvement was reported in 10 (50%) of the patients. These results are in accordance with patients’ subjective rating of global impression of change in our study because 10 (50%) patients in the intervention group rated their overall situation as clinically satisfactorily improved compared to 0 patients in the control group. Thus, it seems that EMDR treatment for nsCBP-t, which directly focuses on disturbing pain-related memories, associated memories, current pain perception, or anticipated stressful situations, may be a new, promising, and emerging treatment that is suitable for nsCBP-t patients.

To our knowledge, except a small case series (29), the current study is the first to suggest that EMDR-based treatment may successfully reduce pain intensity and pain-related disability in patients with nsCBP-t. The improvements in our study occurred despite preceding, long-standing histories of treatment-refractory pain. Moreover, our data indicated that EMDR treatment seems to be a safe and brief approach.

Although a clinically relevant improvement was suggested in about 50% of the patients in the intervention group, this was not the case for the other 50%. In a larger randomized controlled trial, prognostic variables for responders should be identified to foster the allocation of patients to treatment that is appropriate for them. Because existing treatments only have low to moderate effects (5–7) and fail to meet patients’ success criteria (8, 9), our results are promising because the treatment seemed to meet patients’ success criteria and clinically relevant changes were suggested for half of the treated patients. However, due to our pilot study design, results should be interpreted with caution.

Explorative 6-month follow-up suggests maintained improvements in pain intensity by EMDR intervention. The effect was smaller than directly after treatment, what might be the case because almost half of the patients in the control group started psychotherapeutic treatment during follow-up. The moderate effect for disability, however, attenuated to small or negligible size. These findings suggest the implementation of subsequent booster sessions that might be helpful to sustain the achieved effects after treatment. Considering clinical relevance on individual patient basis, after 6-month follow-up, 13 (65%) and 7 (35%) of the patients in the intervention group showed relevant improvement in pain intensity and disability, respectively. Eight (40%) of the patients in the intervention group rated the improvement as clinically relevant from their perspective. This suggests that treatment might be especially successful in a subgroup of nsCBP-t patients who should be identified in future research.

The preliminary hints for the success of EMDR treatment for some of the nsCBP-t patients give rise to the question of what mechanisms are responsible for these effects. Several possible explanations for the mechanism underlying pain reduction by EMDR are discussed. Recent studies show that in the context of pain chronification in the brain, there is a shift away from the classic pain-processing regions of the brain and toward the emotional networks of the brain (21). EMDR, as an established procedure for exposing emotional response from trauma therapy, is suggested to specifically process this dysfunctional emotional shift (49, 50). In addition to this, EMDR-specific elements of desensitization and reprocessing of emotional distress induce some psychophysiological de-arousal (51, 52). EMDR also contains numerous other pain-relief therapeutic elements that are not specific to EMDR [e.g., exposure, relaxation, and hypnotic techniques as well as improved coping abilities (28, 53)]. The assumption of an improved ability to cope with pain is consistent with the observed reduction of pain-related disability in this study. Therefore, more research on the involved mechanisms and their convertibility by treatment is necessary to examine the question of underlying mechanisms.

However, there are some potential study limitations that might have biased our results. First, as common for pilot studies, the study was not sufficiently powered for confirmatory decisions about efficacy of EMDR in nsCBP-t patients. Moreover, EMDR was not compared to other psychotherapeutic treatments. However, these limitations were accepted fitting with our proof of concept pilot RCT design that was not confirmatory but aimed at a first impression of potential effects of EMDR in nsCBP-t. Therefore, these first results considering EMDR in nsCBP-t have to be replicated with larger, methodological, and more stringent trials.

Although there are some shortcomings, our study is important because we were the first to use EMDR with nsCBP-t patients and applied a more stringent research design than previous studies (e.g., homogeneous patient groups, randomization, control group, standardized treatment protocols). The effects reported in our study suggested also that EMDR treatment may satisfy patients’ success criteria and may offer sufficient pain relief for nsCBP-t patients. Moreover, although patients reported previous traumatic events, treatment was mainly pain focused. Although our results are preliminary and should be interpreted with caution, our study will facilitate research on this topic.

## Conclusion

Our study suggests that our EMDR manual may be safe and seems to achieve clinically relevant reductions in pain intensity and disability in nsCBP-t patients who seem to be rated relevant by the patients. For pain intensity, the effect decreased within 6-month follow-up but was suggested to be further moderate and relevant. Thus, EMDR may be a promising treatment for nsCBP-t. Nevertheless, these preliminary results must be interpreted with caution. In the next step, a methodologically more stringent RCT on EMDR in nsCBP-t with an appropriate sample size and a psychosocial comparator intervention is necessary to underpin and confirm our findings. If so, in addition, identification of responders and non-responders is desirable to allocate patients to appropriate treatments. The results will be important in determining whether routine use of EMDR therapy for nsCBP-t and nsCBP patients in general is indicated.

## Author Contributions

AG contributed substantially to conception and design of the work, analyzed and interpreted the data, and drafted the manuscript. GS, SJ, and SL were involved in acquisition and interpretation of data and revised the manuscript critically for important intellectual content. MH contributed substantially to conception and design of the work, interpreted the data, and revised the manuscript critically for important intellectual content. JT contributed substantially to conception and design of the work, was involved of acquisition and interpretation of data, and drafted the manuscript. All the authors approved the final version to be published and agreed to be accountable for all aspects of the work in ensuring that questions related to the accuracy or integrity of any part of the work are appropriately investigated and resolved.

## Conflict of Interest Statement

The authors declare that the research was conducted in the absence of any commercial or financial relationships that could be construed as a potential conflict of interest.
